# CeO_2_ Nanoparticle-Loaded MnO_2_ Nanoflowers for Selective Catalytic Reduction of NO_x_ with NH_3_ at Low Temperatures

**DOI:** 10.3390/molecules27154863

**Published:** 2022-07-29

**Authors:** Shun Li, Zuquan Zheng, Zhicheng Zhao, Youling Wang, Yao Yao, Yong Liu, Jianming Zhang, Zuotai Zhang

**Affiliations:** 1 Institute of Quantum and Sustainable Technology (IQST), School of Chemistry and Chemical Engineering, Jiangsu University, Zhenjiang 212013, China; shun@ujs.edu.cn; 2 Guangdong Provincial Key Laboratory of Soil and Groundwater Pollution Control, School of Environmental Science and Engineering, Southern University of Science and Technology, Shenzhen 518055, China; zuquanz@163.com; 3 Foshan (Southern China) Institute for New Materials, Foshan 528200, China; zhaozhicheng@fscinm.com (Z.Z.); yaoyao@fscinm.com (Y.Y.); 4 Shunde Graduate School, University of Science and Technology Beijing, Foshan 528399, China; youlwang@163.com

**Keywords:** Mn-Ce mixed oxide, ion-adsorption, selective catalytic reduction, NO_x_ conversion

## Abstract

CeO_2_ nanoparticle-loaded MnO_2_ nanoflowers, prepared by a hydrothermal method followed by an adsorption-calcination technique, were utilized for selective catalytic reduction (SCR) of NO_x_ with NH_3_ at low temperatures. The effects of Ce/Mn ratio and thermal calcination temperature on the NH_3_–SCR activity of the CeO_2_-MnO_2_ nanocomposites were studied comprehensively. The as-prepared CeO_2_-MnO_2_ catalysts show high NO_x_ reduction efficiency in the temperature range of 150–300 °C, with a complete NO_x_ conversion at 200 °C for the optimal sample. The excellent NH_3_–SCR performance could be ascribed to high surface area, intimate contact, and strong synergistic interaction between CeO_2_ nanoparticles and MnO_2_ nanoflowers of the well-designed composite catalyst. The in situ diffuse reflectance infrared Fourier transform spectroscopy (DRIFTs) characterizations evidence that the SCR reaction on the surface of the CeO_2_-MnO_2_ nanocomposites mainly follows the Langmuir–Hinshelwood (L-H) mechanism. Our work provides useful guidance for the development of composite oxide-based low temperature NH_3_–SCR catalysts.

## 1. Introduction

With the fast developing modern industrialization, nitrogen oxides (NO_x_) emitted from industrial production and automobile exhaust have become one of the major environmental pollution issues, which may cause acid rain and greenhouse effects [1,2]. Long-term exposure to NO_x_ can cause several side effects to human health, such as reduced lung function, increased risk of respiratory conditions, and increased response to allergens. As an eco-friendly and efficient approach, selective catalytic reduction of NO_x_ with NH_3_ (NH_3_–SCR) is widely used for removing NO_x_ [3,4,5,6,7]. The core of the NH_3_–SCR technology is the catalyst. Although V_2_O_5_-WO_3_(MoO_3_)/TiO_2_ catalysts have been utilized commercially in the temperature range of 300–400 °C [8], the increasing demands for outstanding catalytic activity at low working temperature with high stability have stimulated the development of novel SCR catalysts.

During the past few decades, enormous research work has been carried out to exploit mixed metal oxides NH_3_–SCR catalysts composed of TiO_2_, VO*_x_*, MnO*_x_*, CeO_2_, Fe_2_O_3_, ZrO_3_ or WO_3_ etc., owing to their high stability, strong synergistic interaction, excellent oxygen storage capacity, and redox ability [9,10,11,12,13,14]. Among all, CeO_2_-MnO_x_ composites with outstanding low-temperature activity, excellent oxygen storage, and release capacity, and strong mechanical strength, have received considerable attention as a promising candidate NH_3_–SCR catalyst [15,16,17,18,19,20]. Nevertheless, CeO_2_-MnO_x_ composite catalysts still suffer from several deficiencies in practical applications due to their poor thermal stability, low reduction efficiency, and low sulfur resistance. Considering that the architecture of composite with close contact between each component is crucial for promoting the SCR catalytic activity, considerable work have been conducted to the design and fabrication of hierarchical CeO_2_-MnO_x_ composites at nanoscale. For instance, it has been reported that CeO_2_-MnO_x_ mixed oxides with hollow structures possess excellent low temperature NO_x_ storage capacity and NH_3_–SCR activity owing to the large surface area, rich active sites, and confined micro-environment [21,22,23,24]. Therefore, the development of well-designed CeO_2_-MnO_x_ composite catalyst with desirable intimate contact at nanoscale is of importance for further enhancing their NH_3_–SCR catalytic performance.

**Scheme 1:**
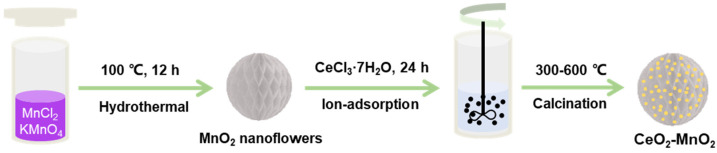
Schematic of the synthesis process for CeO_2_-MnO_2_ nanocomposites.

In this work, a rational designed CeO_2_ nanoparticle-loaded MnO_2_ nanoflowers (NFs) with adjustable Ce/Mn ratios were prepared through combining a simple hydrothermal and subsequent ion-adsorption-calcination method, as illustrated in Scheme 1. A variety of physical and chemical characterization methods were used to investigate the phase composition, microstructure, and surface characteristics. The unique architecture of the CeO_2_-MnO_2_ nanocomposites consisting of MnO_2_ nanoflowers loaded with ultrasmall CeO_2_ nanoparticles allows the composite with high surface area, intimate contact, and strong synergistic interaction between CeO_2_ and MnO_2_. The catalysts displayed excellent low-temperature NH_3_–SCR activity for NO_x_ reduction, obtaining a nearly 100% conversion efficiency at 200 °C. Moreover, the NH_3_–SCR reaction mechanism was revealed by in situ diffuse reflectance infrared Fourier transform spectroscopy (DRIFTs).

## 2. Experimental

### 2.1. Materials

The chemicals of CeCl_3_·7H_2_O (AR, ≥99.9%), MnCl_2_·4H_2_O (AR, ≥99.99%), and KMnO_4_ (AR, ≥99.5%) were purchased from Aladdin Reagents and used as received without any further purification. Water was deionized (D.I. water) to reach the Nanopure grade (18.2 MΩ∙cm at 25 °C).

### 2.2. Synthesis of MnO_2_ NFs

MnO_2_ NFs were synthesized using a hydrothermal method. Typically, 0.5 g KMnO_4_ was dissolved in 50 mL of D.I. water and stirred for 15 min. After that, 1 mol/L MnCl_2_·4H_2_O solution was added into the above solution and stirred for 1 h. Then the mixed solution was transferred into a 100 mL Teflon-lined stainless-steel autoclave and heated in a conventional oven at 100 °C for 12 h. After cooling down to room temperature, the as-obtained powder was washed with D.I. water for three times and dried at 60 °C.

### 2.3. Synthesis of CeO_2_-MnO_2_ Nanocomposites

Typically, 0.1 g of the as-synthesized MnO_2_ NFs powder was ultrasonically dispersed in 100 mL of aqueous solution containing certain amount of CeCl_3_·7H_2_O (0.02, 0.04, 0.06, and 0.08 g), denoted as Ce/Mn-1, Ce/Mn-2, Ce/Mn-3, and Ce/Mn-4, respectively. Subsequently, the suspension was magnetically stirred for 24 h to ensure the complete adsorption of the Ce ions onto the MnO_2_ NFs. Then the samples were collected after washing with D.I. water and dried at 60 °C for 12 h. Finally, the as-obtained sample was calcined at different temperatures (300 to 600 °C) for 2 h.

### 2.4. Materials Characterizations

The crystal structure was investigated by powder X-ray diffraction (XRD) with a Bruker D8 Advance diffractometer (Bruker, Karlsruhe, Germany) with CuKα radiation. The morphology and microstructure were studied by field emission scanning electron microscopy (SEM, Zeiss Merlin, Carl Zeiss NTS GmbH, Oberkochen, Germany) and transmission electron microscopy (TEM, FEI Talos F200X , Thermo Fisher Scientific, Hillsboro, OR, USA), equipped with an energy-dispersive X-ray spectroscope (EDS). X-ray photoelectron spectroscopy (XPS) characterizations were performed using a PHI 5600 XPS system (Perkin-Elmer, Waltham, MA, USA). The obtained spectra were calibrated with the C1s peak at 284.6 eV. The specific surface areas and pore size distributions were estimated by the Brunauer–Emmett–Teller (BET) and Barrett–Joyner–Halenda (BJH) methods with N_2_ adsorption and desorption isotherms obtained on a Micrometrics ASAP 2020 system. 

### 2.5. NH_3_–SCR Activity Measurement

The SCR activity of the catalysts was evaluated using a conventional fixed-bed quartz microreactor operating in a steady state flow mode under atmospheric pressure. In a typical test, the as-obtained catalysts (50 mg) and quartz sand (200 mg) were mixed and packed between two quartz glass wool plugs. The simulated flue gas composition was NO (500 ppm), NH_3_ (500 ppm), O_2_ (2%), and balance gas N_2_. The total flow rate was 100 mL min^−1^, which corresponds to a gas hourly space velocity (GHSV) of 16,000 h^−1^. The concentrations of NO were measured by a chemiluminescence NO_x_ analyzer (42i-HL, Thermo Scientific). The NO conversion rate was calculated by the following equation:

(1) $${\mathrm{NO}}_{\mathrm{conversion}}=\frac{{\left[\mathrm{NO}\right]}_{\mathrm{in}}{\;-\;[\mathrm{NO}]}_{\mathrm{out}}}{{\left[\mathrm{NO}\right]}_{\mathrm{in}}}\;\text{×}\;100\%$$

## 3. Results and Discussion

### 3.1. Effect of CeO_2_ and MnO_2_ Ratio

**Figure 1:**
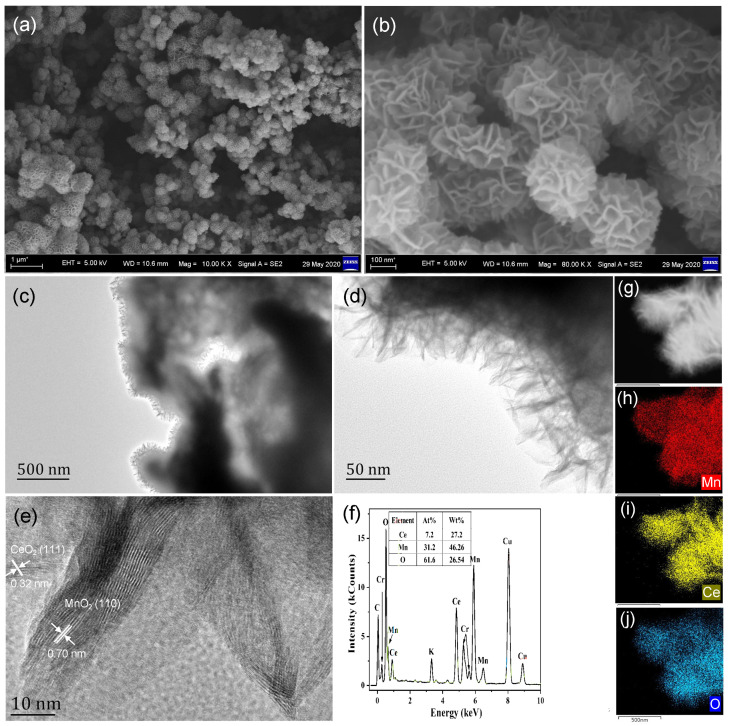
(**a**,**b**) SEM, (**c**) and (**d**) TEM, (**e**) HRTEM, (**f**) EDS spectrum, (**g**) HAADF-STEM, and (**h**–**j**) mapping images of Ce/Mn-3 (300).

The CeO_2_-MnO_2_ nanocomposites with different Ce/Mn ratios were synthesized first. The morphology of the catalysts (taking Ce/Mn-3 as a representative) was characterized by SEM and TEM. As shown in the SEM images (Figure 1a,b), the as-prepared CeO_2_-MnO_2_ catalyst possesses flower-like morphology with sizes of about 200–300 nm. The TEM images (Figure 1c,d,g) clearly display that the nanoflowers are composed of numerous sheets with thickness of several nanometers. From the HRTEM image (Figure 1e), the interplanar spacings were measured to be 0.32 and 0.70 nm, which can be assigned to the (111) plane of CeO_2_ and (001) plane of δ-MnO_2_, respectively. The EDS spectrum (Figure 1f) confirms that the composites contain three elements of Ce, Mn, and O, with atomic content of 7.2%, 31.2%, and 61.6%, respectively. Meanwhile, the EDS mapping diagrams (Figure 1h–j) clearly display the homogenous distribution of Mn, Ce, and O elements throughout the NFs, demonstrating the uniform growth of CeO_2_ nanoparticles on the MnO_2_ NFs. Such a uniform distribution is highly expected to promote their interaction and synergistic effect of Ce and Mn mixed oxides during the catalytic reactions [24,25].

**Figure 2:**
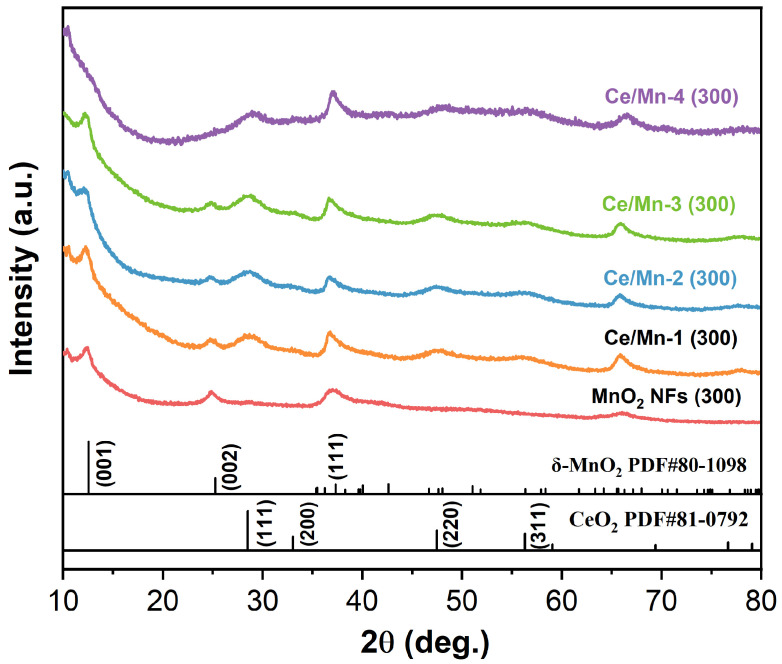
XRD patterns of MnO_2_ NFs and CeO_2_-MnO_2_ composites with different Ce/Mn ratios calcinated at 300 °C.

The crystal structure of the CeO_2_-MnO_2_ catalysts with different Ce and Mn ratios after thermal treatment at 300 °C was analyzed by XRD. As shown in Figure 2, all diffraction peaks of the pristine MnO_2_ sample could be well indexed to δ-MnO_2_ crystal structure (JCPDS No. 80-1098) with predominant crystal planes of (001), (002), and (111). After the adsorption of Ce^3+^ and the following calcination treatment, the diffraction peaks corresponding to CeO_2_ (JCPDS No. 81-0792) appeared with predominant crystal planes of (111), (200), and (220), demonstrating the formation of CeO_2_-MnO_2_ composites. The peak intensity of CeO_2_ gradually increases with the increasing CeCl_3_·7H_2_O content. The broad diffraction peaks suggest the small crystal size of the loaded CeO_2_, which is consistent with the TEM observations.

**Figure 3:**
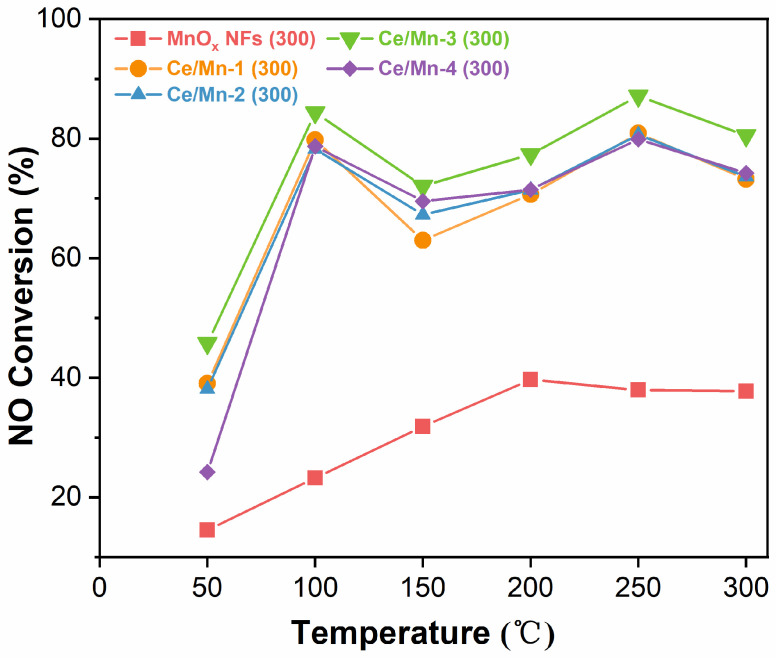
NO conversion curves of MnO_2_ NFs and CeO_2_-MnO_2_ catalysts with different Ce/Mn ratios.

The NH_3_–SCR activity of the as-prepared catalysts calcinated at 300 °C was evaluated. As shown in Figure 3, all the CeO_2_-MnO_2_ composite catalysts with different Ce and Mn ratios exhibit much higher activity in a relatively wide range of temperature from 50 to 300 °C, compared to pristine MnO_2_. As the Ce content increases, the NO conversion rate increases significantly, while decreases at higher Ce contents. Among all samples, Ce/Mn-3 (300) shows the optimal NH_3_–SCR catalytic performance, reaching the highest NO conversion rate of about 87.6% at 250 °C.

**Figure 4:**
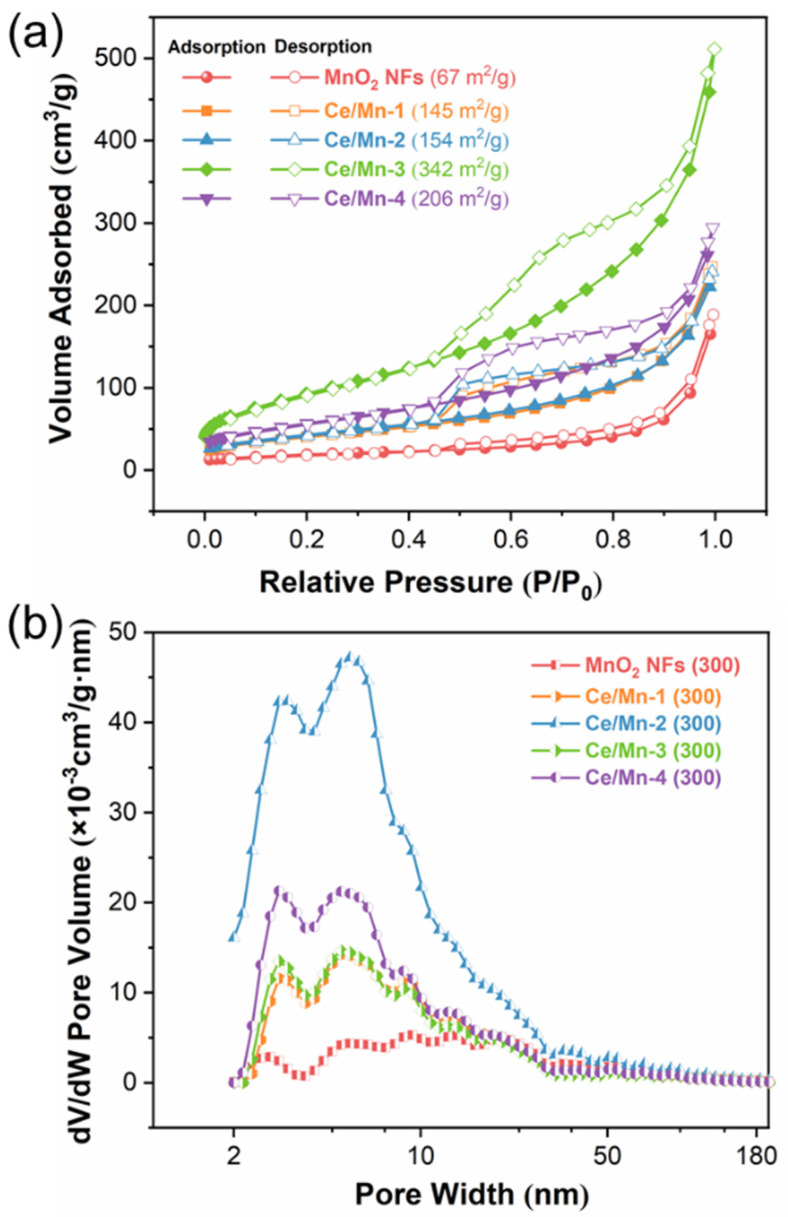
(**a**) N_2_ adsorption–desorption isotherm and (**b**) pore size distribution of MnO_2_ NFs and CeO_2_-MnO_2_ catalysts with different Ce/Mn ratios.

The specific surface areas (SSAs) and pore features are crucial factors determining the catalytic activity. As shown in Figure 4a, the N_2_ adsorption–desorption isotherms of the as-prepared catalysts exhibit type IV isotherm with H3 hysteresis loops, demonstrating their mesoporous structure. The CeO_2_-MnO_2_ composite catalysts possess significantly higher SSAs in comparison to the pristine MnO_2_, estimated by the BET method. The measured SSAs follow the order of Ce/Mn-3 (342 m^2^ g^−1^) > Ce/Mn-4 (206 m^2^ g^−1^) > Ce/Mn-2 (154 m^2^ g^−1^) > Ce/Mn-1 (145 m^2^ g^−1^) > MnO_2_ (67 m^2^ g^−1^). Figure 4b displays the pore size distribution curves estimated by the BJH method. For all samples, the pore size is mostly distributed in the range of 4 to 10 nm, which belongs to the mesoporous structure. The SSAs for the as-prepared CeO_2_ loaded MnO_2_ NFs are significantly higher than most of the reported CeO_2_-MnO_x_ nanocomposite catalysts [26,27,28]. The large SSAs and porous features of the as-synthesized CeO_2_-MnO_2_ catalysts (e.g., Ce/Mn-3 (300)) with more catalytic active sites show great advantages for the adsorption and diffusion of gaseous reactants toward high NH_3_-SCR activity [29].

### 3.2. Effect of Thermal Treatment Temperature

**Figure 5:**
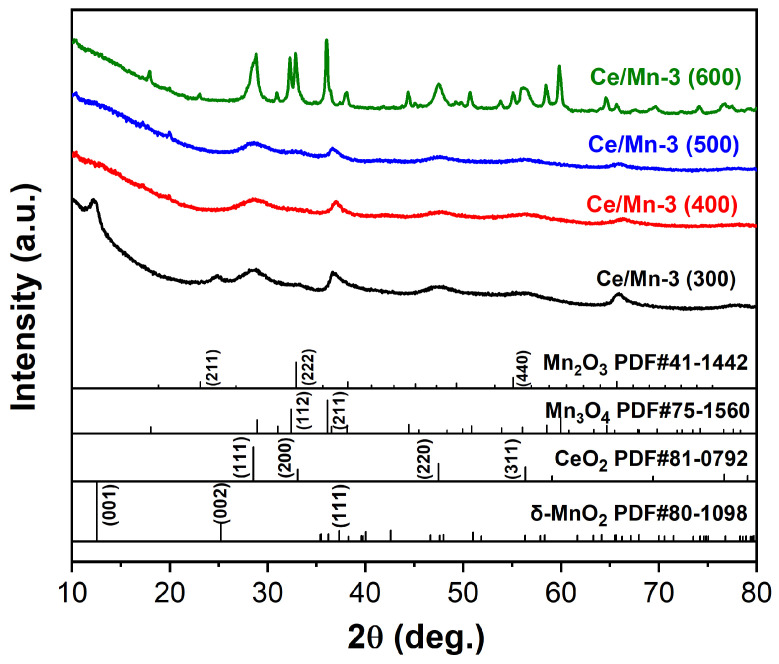
XRD patterns of the Ce/Mn-3 catalysts calcinated at different temperatures.

To further optimize the NH_3_–SCR catalytic activity of the CeO_2_-MnO_2_ composite catalysts, the effect of thermal treatment temperature was investigated, selecting the Ce/Mn-3 sample with the highest activity. Figure 5 displays the XRD patterns of Ce/Mn-3 catalysts after calcinating at four different temperatures of 300 °C, 400 °C, 500 °C, and 600 °C. The samples of Ce/Mn-3 (300), Ce/Mn-3 (400), and Ce/Mn-3 (500) exhibit consistent diffraction peaks, which can be indexed to δ-MnO_2_ (JCPDS No. 80-1098) with predominant crystal planes of (001), (002) and (111) and CeO_2_ (JCPDS No. 81-0792) with predominant crystal planes of (111), (200), and (220). For the sample treated under 600 °C, secondary phases of Mn_2_O_3_ (JCPDS No. 89-2809) and Mn_3_O_4_ (JCPDS No. 75-1560) appeared.

**Figure 6:**
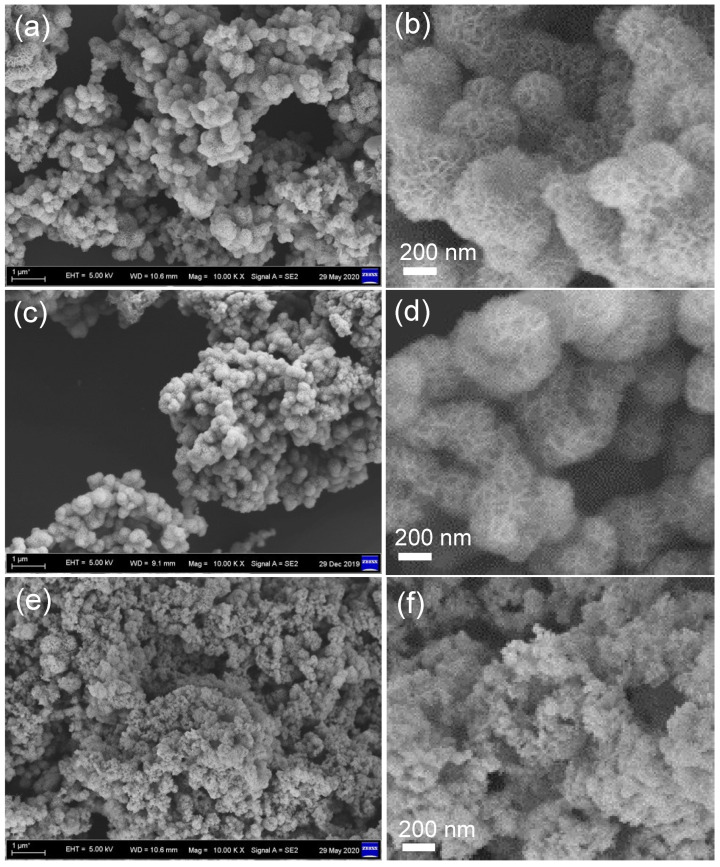
SEM images of the Ce/Mn-3 catalysts calcinated at different temperatures: (**a**) and (**b**) 400 °C, (**c**) and (**d**) 500 °C, and (**e**) and (**f**) 600 °C.

The SEM images of Ce/Mn-3 catalysts after thermal treatment at different temperatures are displayed in Figure 6. It is clearly shown that spherical flower-like morphology structure was well maintained when the calcination temperature was under 500 °C (Figure 6a–d), compared with that of Ce/Mn-3 (300). Nevertheless, the flower-like hierarchical microstructure of the catalyst for Ce/Mn-3 (600) sample disappeared, forming irregular shaped particles (Figure 6e,f).

**Figure 7:**
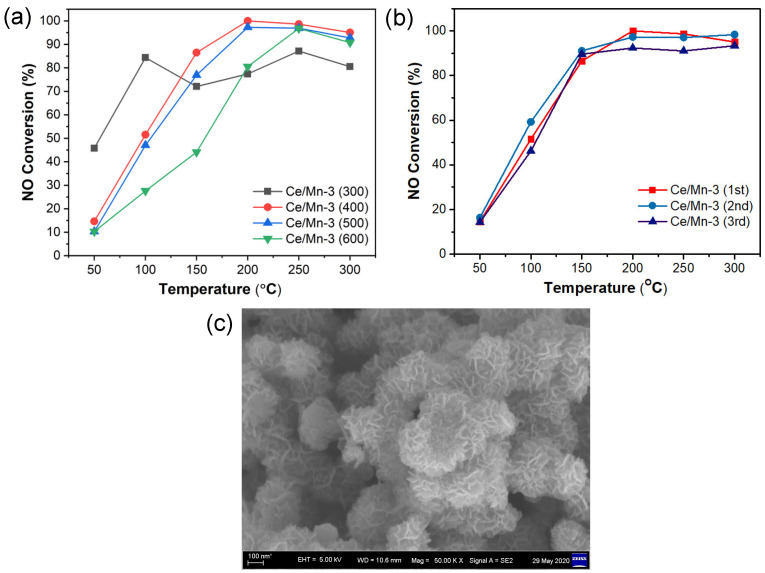
(**a**) NO conversion curves of the Ce/Mn-3 catalysts calcinated at different temperatures, (**b**) recycling catalytic activity tests of Ce/Mn-3 (400), and (**c**) SEM image of Ce/Mn-3 (400) after catalytic reactions.

Subsequently, the NH_3_–SCR activity of different as-prepared Ce/Mn-3 catalysts were tested. As shown in Figure 7, in the range of 50~200 °C, the NO conversion rate of the catalysts (except for Ce/Mn-3 (300)) increases with the increasement of thermal treatment temperature. Among all the catalysts, Ce/Mn-3 (400) exhibited the highest SCR performance in the low-temperature window from 150 to 300 °C, reaching a 100% removal rate of NO at 200 °C. The activity declined for the catalysts treated at higher temperatures (above 500 °C), which could be ascribed to the changes of structural and surface characteristics of the catalyst, discussed as follows.

**Figure 8:**
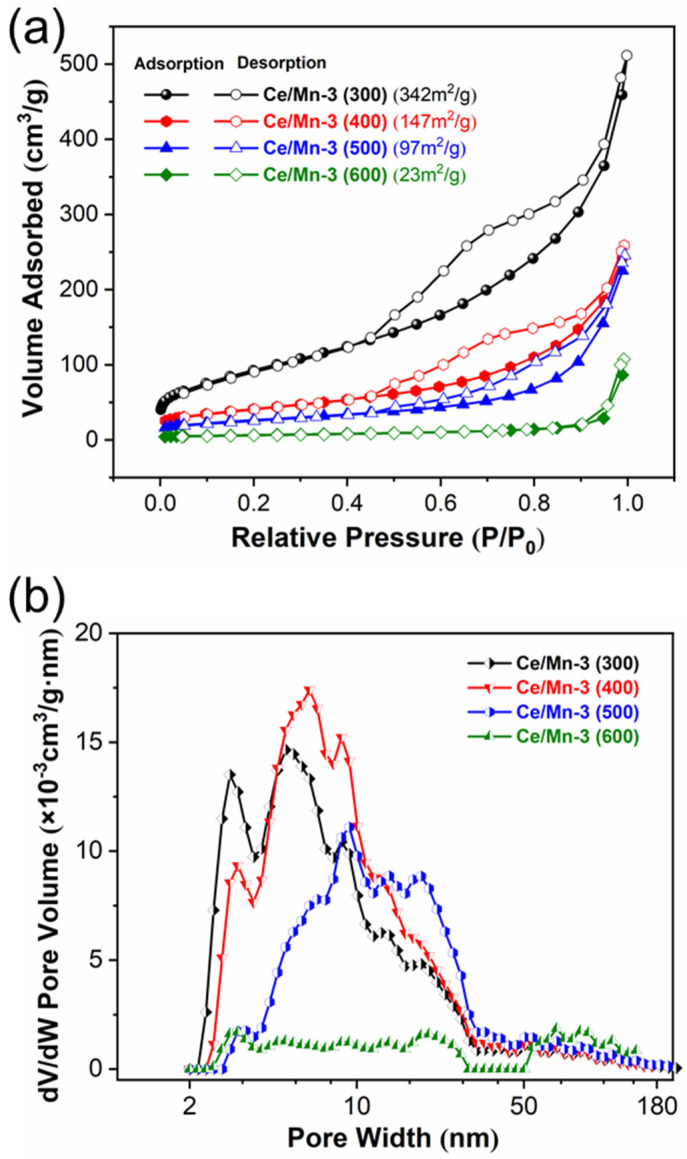
(**a**) N_2_ adsorption–desorption isotherm and (**b**) pore size distribution of Ce/Mn-3 catalysts calcinated at different temperatures.

Figure 8 exhibits the N_2_ adsorption–desorption isotherms and the pore size distributions of various Ce/Mn-3 catalysts calcinated at different temperatures. From Figure 8a, all the catalysts display IV type N_2_ isotherm with H3 type loops, which correspond to the mesoporous structure. Among all samples, the Ce/Mn-3 catalyst prepared at 300 °C has the largest SSA. The SSAs follow the order of Ce/Mn-3 (300) (342 m^2^ g^−1^) > Ce/Mn-3 (400) (147 m^2^ g^−1^) > Ce/Mn-3 (500) (97 m^2^ g^−1^) > Ce/Mn-3 (600) (23 m^2^ g^−1^). Figure 8b displays the pore size distribution curves estimated by the BJH method, demonstrating that the mesopores are mainly distributed from 4 to 20 nm.

**Figure 9:**
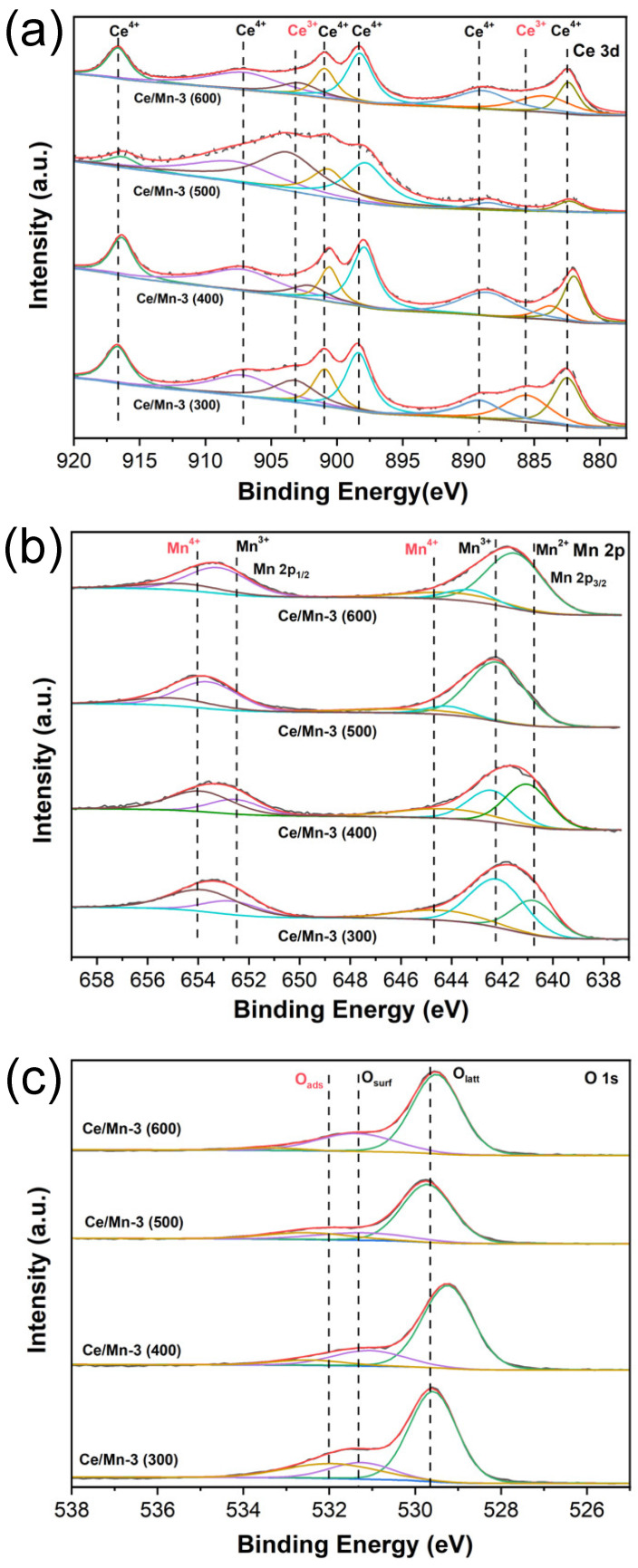
High-resolution XPS spectra of Ce/Mn-3 catalysts calcinated at different temperatures: (**a**) Ce 3d, (**b**) Mn 2p, and (**c**) O 1s.

XPS spectra were measured to analyze the element oxidation states of the composite catalysts calcinated at different temperatures. Figure 9 shows the high-resolution Ce 3d, Mn 2p, and O 1s XPS spectra. As displayed in Figure 9a, the peaks of Ce^3+^ are located at ~885.7 eV and ~903.1 eV, while the remaining peaks are attributed to Ce^4+^ [30,31,32]. As listed in Table 1, the calculated Ce^3+^ content (24.84%) in Ce/Mn-3 (400) is the highest among all CeO_2_-MnO_2_ samples, which is beneficial for the adsorption of NH^4+^ [33]. In addition, the Mn 2p 3/2 peaks in Figure 9a can be fitted into three peaks, which belong to Mn^2+^ (640.7~641.5 eV), Mn^3+^ (642.2~643.5 eV), and Mn^4+^ (644.8 eV), respectively [34,35]. Generally, Mn with high oxidation states (Mn^4+^) can promote the oxidation performance of NO over CeO_2_-MnO_2_ catalysts at low temperatures. The Ce/Mn-3 (400) catalyst contains more Mn^4+^ (38.55%) than other samples (Table 1), indicating that the catalyst may exhibit outstanding NH_3_-SCR activity at low-temperature owing to the excellent oxidation ability. Figure 9c shows the XPS spectra of O 1s, which could be deconvoluted into lattice oxygen O_latt_ (~529.6 eV), surface oxygen O_surf_ (~531.3 eV), and chemically adsorbed oxygen O_ads_ (~532.1 eV) [36]. As listed in Table 1, the Ce/Mn-3 sample contains the largest ratio of O_ads_ (23.83%), which has great advantage for the SCR reaction [27,37]. These results are well consistent with the NH_3_–SCR activity of different catalysts as discussed above.

### 3.3. In Situ DRIFTs Studies

**Figure 10:**
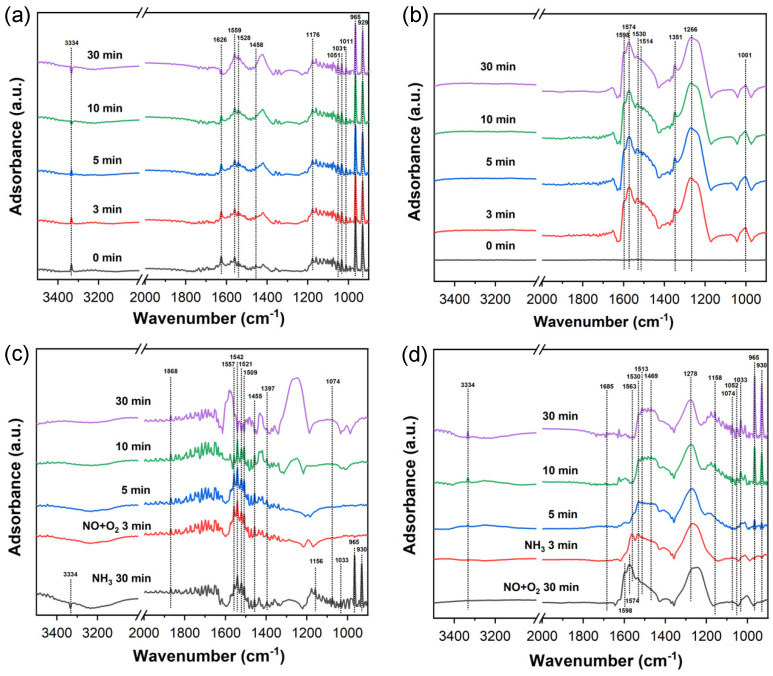
In situ DRIFTs of Ce/Mn-3 (300) at 200 °C over different time: (**a**) NH_3_ adsorption, (**b**) NO + O_2_ adsorption, (**c**) reaction of pre-adsorbed NH_3_ with NO + O_2_, and (**d**) reaction of pre-adsorbed NO with NH_3_.

To disclose the NH_3_–SCR reaction mechanism over the CeO_2_-MnO_2_ composite catalysts, in situ DRIFTs analysis was conducted by tracking the changes in the adsorbed species on the catalyst (taking Ce/Mn-3 (300) as a representative) surface during the reaction process. Figure 10a shows the NH_3_ adsorption DRIFT spectra at 200 °C over the CeO_2_-MnO_2_ composite catalyst. The bands at 1692, 1642, and 1458 cm^−1^ belong to the NH^4+^ adsorbed on the Brønsted acid site, while the peaks at 3334, 1626, and 1040~1200 cm^−1^ belong to the NH_3_ adsorbed on the Lewis acid site [17,38,39]. In addition, the peaks at 992, 965, and 930 cm^−1^ represent weakly adsorbed gaseous NH_3_ on the catalyst’s surface. As increasing the adsorption time, the peak at 1458 cm^−1^ belonging to the Brønsted acid site gradually disappeared, indicating that the surface NH_3_ adsorption is dominated by the Lewis acid site. The DRIFT spectra of NO + O_2_ adsorption on the CeO_2_-MnO_2_ composite catalyst along with the time at 200 °C are presented in Figure 10b. The peaks at 1574, 1530, and 1514 cm^−1^ belonging to nitrates weakened after NO and O_2_ were introduced for 30 min, indicating the generation of intermediate products released from the active site to produce N_2_ and H_2_O [40].

The in situ DRIFT spectra of the CeO_2_-MnO_2_ catalyst with NO + 5% O_2_ reacted with the pre-adsorbed NH_3_ at 200 °C are displayed in Figure 10c. Notably, all vibration bands belonging to the adsorbed ammonia species (NH_3_ and NH^4+^) vanished rapidly after 3 min after introducing NO and O_2_, demonstrating the reaction with different ammonia species. The peak at 1455 cm^−1^ vanished in 30 min, and a new adsorption peak located at 1557 cm^−1^ assigned to the bipedal nitrate was observed. The peak at 1397 cm^−1^ could be ascribed to the intermediate species from the surface adsorbed NH_3_ and NO_x_ species. As shown in Figure 10d, the in situ DRIFT spectra were further measured to reveal the reaction of NH_3_ with pre-adsorbed NO on the surface of the CeO_2_-MnO_2_ catalyst at 200 °C. After introducing NH_3_, the vibration peaks of weakly adsorbed NO_2_ at 1598 and 1574 cm^−1^ on the surface of Ce/Mn-3 disappeared rapidly. In addition, new vibration peaks at 1685 and 1469 cm^−1^ owing to the adsorption of NH^4+^ at the Brønsted acid site were observed. Additionally, a new adsorption peak at 1513 cm^−1^ was also detected, which can be ascribed to the bidentate nitrate species resulting from the oxidation of NH_3_ by O_2_ [9]. Based on the above analysis, we can conclude that the Langmuir–Hinshelwood (L-H) may play an important role in the NH_3_–SCR reactions over the CeO_2_-MnO_2_ composite catalysts because of the formation of a larger amount of absorbed NH_3_ (NH^4+^), NO_2_, and NH_2_ intermediate [9,41].

Moreover, further studies were carried out to reveal the effect of SO_2_ and H_2_O on the NH_3_–SCR catalytic activity of the CeO_2_-MnO_2_ composite catalyst. The results indicated that the deactivation of the catalyst with the presence of H_2_O can be ascribed to the competitive adsorption between NH_3_ and H_2_O on the Lewis acid sites, which is a reversible process. In the case of SO_2_, it will lead to permanent catalyst poisoning through the sulfation of surface active sites. In addition, the competitive adsorption of NO and SO_2_ as well as the formation of (NH_4_)_2_SO_4_ on the catalysts’ surface may also contribute to the partial deactivation of the CeO_2_-MnO_2_ catalyst. These conclusions are consistent with previous reports [42]. To regenerate the deactivated CeO_2_-MnO_2_ catalyst, water washing and thermal or reductive regeneration methods can be used. Further works are still needed to clarify the mechanism of poisoning effect of SO_2_ and H_2_O and corresponding regeneration methods.

## 4. Conclusions

In summary, we have synthesized a novel CeO_2_-MnO_2_ composite catalyst composed of CeO_2_ nanoparticles grown on MnO_2_ nanoflowers by combining hydrothermal method and ion-adsorption-calcination technique. The unique flower-like hierarchical microstructure and high specific surface area endow the catalysts with desirable intimate contact and synergistic effect, which leads to excellent NH_3_–SCR NO_x_ reduction activity in a wide temperature window from 150 to 300 °C, obtaining a maximal NO conversion of nearly 100% at 200 °C. Moreover, the high contents of Ce^3+^, Mn^4+^, and surface O_ads_ in the CeO_2_-MnO_2_ catalyst could be also responsible for their excellent catalytic performance. Furthermore, in situ DRIFT experiments reveal that the NH_3_–SCR reaction route on the surface of the CeO_2_-MnO_2_ hybrid catalysts is dominated by the L-H mechanism. The as-obtained CeO_2_-MnO_2_ composites in this work have great prospects for NH_3_-SCR reaction of NO_x_ at low temperatures.

## Figures and Tables

**Table 1:** The calculated ratios of Ce^3+^, Mn^4+^, and O_ads_ in different catalysts based on XPS analysis.

	Ce^3+^/Ce (%)	Mn^4+^/Mn (%)	O_ads_/O (%)
Ce/Mn-3 (300)	23.79	34.4	19.8
Ce/Mn-3 (400)	24.84	38.55	23.83
Ce/Mn-3 (500)	11.8	17.32	6.47
Ce/Mn-3 (600)	18.94	10.1	2.89

## Data Availability

Data is contained within the article.
